# BCL11B Is Up-Regulated by EWS/FLI and Contributes to the Transformed Phenotype in Ewing Sarcoma

**DOI:** 10.1371/journal.pone.0059369

**Published:** 2013-03-19

**Authors:** Elizabeth T. Wiles, Bianca Lui-Sargent, Russell Bell, Stephen L. Lessnick

**Affiliations:** 1 Department of Oncological Sciences, University of Utah, Salt Lake City, Utah, United States of America; 2 Center for Children’s Cancer Research, Huntsman Cancer Institute, Salt Lake City, Utah, United States of America; 3 Division of Pediatric Hematology/Oncology, University of Utah, Salt Lake City, Utah, United States of America; Tulane University School of Medicine, United States of America

## Abstract

The EWS/FLI translocation product is the causative oncogene in Ewing sarcoma and acts as an aberrant transcription factor. EWS/FLI dysregulates gene expression during tumorigenesis by abnormally activating or repressing genes. The expression levels of thousands of genes are affected in Ewing sarcoma, however, it is unknown which of these genes contribute to the transformed phenotype. Here we characterize BCL11B as an up-regulated EWS/FLI target that is necessary for the maintenance of transformation in patient derived Ewing sarcoma cells lines. BCL11B, a zinc finger transcription factor, acts as a transcriptional repressor in Ewing’s sarcoma and contributes to the EWS/FLI repressed gene signature. BCL11B repressive activity is mediated by the NuRD co-repressor complex. We further demonstrate that re-expression of SPRY1, a repressed target of BCL11B, limits the transformation capacity of Ewing sarcoma cells. These data define a new pathway downstream of EWS/FLI required for oncogenic maintenance in Ewing sarcoma.

## Introduction

Ewing sarcoma is an aggressive tumor that occurs in the bone and soft tissue of the pediatric and young adult population [1]. This tumor is characterized by a chromosomal translocation that fuses the 5′ portion of the *EWSR1* gene (encoding the EWS protein) on chromosome 22, in frame, to the 3′ portion of the *FLI1* gene (encoding the FLI protein) on chromosome 11 [2]. The resulting fusion protein, EWS/FLI, retains the ETS-type DNA binding domain from FLI as well as the transcriptional activating and repressing domains from EWS, and thus acts as an aberrant transcription factor. EWS/FLI is present in ∼85% of Ewing sarcoma cases while the remaining 15% express a fusion between EWS and another member of the ETS family (ERG, ETV1, ETV4, or FEV) [3]. While the cell of origin is unknown, this fusion is thought to occur in a primitive cell type where it prevents terminal differentiation. Initial mutational studies suggest that Ewing sarcoma has a relatively low frequency of alterations in known tumor suppressors or oncogenes, supporting the concept that EWS/FLI, and the genes that it regulates, are largely responsible for oncogenesis and tumor maintenance [4], [5]. Indeed, approaches that reduce the levels of EWS/FLI in Ewing sarcoma cells block the transformed phenotype of these cells [6]–[8].

Microarray analysis of gene expression in Ewing sarcoma cell lines compared to those with reduced EWS/FLI expression reveal significant alterations in the transcriptional signatures. EWS/FLI has been shown to decrease the expression of ∼3000 genes while increasing the mRNA levels of ∼500 genes [9]. It is important to determine which of these dysregulated genes contribute to the transformation process. Several EWS/FLI target genes have previously been identified that contribute to oncogenic processes in Ewing’s sarcoma: *NKX2.2* [7], *NR0B1* [6], *GLI1* [10], *TGFBR2* [11], among others. In this work we characterize B-cell chronic lymphocytic leukemia/lymphoma 11B (*BCL11B*, also known as *CTIP2*), encoding a zinc finger transcription factor [12], as an important target gene up-regulated by EWS/FLI.

BCL11B expression in Ewing sarcoma has been noted previously. *BCL11B* was identified as a gene that was highly expressed in Ewing sarcoma, but only expressed in a restricted subset of normal tissues [13]. More recent microarray studies, including our own, have revealed that BCL11B is induced by EWS/FLI in Ewing sarcoma tumor samples and cell lines [9], [14], as well as two of the proposed cells of origin, mesenchymal stem cells [15] and neural crest stem cells [16]. Interestingly, EWS/FLI does not modulate BCL11B expression in HEK293 cells [13] or NIH3T3 cells [17] (ETW unpublished observation), suggesting that a permissive cellular background is necessary for EWS/FLI to up-regulate BCL11B expression. Chromatin accessibility and transcription factor/co-factor availability may be elements that contribute to these cell specific differences in BCL11B regulation.

*Bcl11b* knock-out mice are initially viable, but die on post-natal day 1 [18]. Phenotypes described in this mouse reveal developmental defects in the skin [19], teeth [20], central nervous system (CNS) [21], [22], and hematopoietic lineage [18], [23]. For example, *Bcl11b* null murine thymocytes fail to undergo T-cell differentiation [23]. In the CNS, Bcl11b is necessary for the connection of corticospinal motor neurons to the spinal cord [22] and for neurogenesis in the dentate gyrus [24]. These observations identify Bcl11b as a pivotal developmental transcription factor involved in fate specification decisions in multiple cell types.

BCL11B has also been studied in the context of malignancies, where it has been described as a haploinsufficient tumor suppressor in T-cell acute lymphoblastic leukemia (T-ALL). *BCL11B* was found to be mutated in 9–16% of human T-ALL samples. [25], [26]. In addition, mouse models of both TLX1 driven T-ALL and gamma-ray induced thymic lymphomas had spontaneous deletions and mutations in *Bcl11b* [26], [27]. These findings, which characterize BCL11B’s tumor inhibitory function, present a conundrum: why does EWS/FLI induce the expression of a postulated tumor suppressor? We therefore sought to define the role of BCL11B in Ewing sarcoma. Surprisingly, in contrast to its tumor suppressive function in leukemia and lymphoma, we now show that BCL11B positively contributes to the transformed phenotype in Ewing sarcoma.

## Materials and Methods

### Constructs and Retroviruses

CHD4 short hairpin RNA (shRNA), EWS/FLI shRNA (EF-2 RNAi), EWS/FLI cDNA and mutants were previously described [7], [28]–[31]. BCL11B shRNA constructs were designed targeting the 3′UTR and cloned into a pMKO.1puro vector [32]. Oligonucleotide sequences for previously unpublished shRNAs are provided in Supplemental Data (Table S1). BCL11B (variant 2) cDNA was sub-cloned from pMIGR [33] into pQCXIN (Clontech). SPRY1 cDNA (Thermo Scientific) was PCR amplified with an amino-terminal 3xFLAG-tag and cloned into pMSCVneo (Clontech).

### Cell Culture

The A673 cell line (American Type Culture Collection) was grown as previously described [34] and TC71 cells (from Timothy Triche, Children’s Hospital Los Angeles) [35] were grown in RPMI supplemented with 10% fetal bovine serum (FBS) and 1% penicillin/streptomycin/glutamine. Following retroviral infection, cells were selected with the appropriate antibiotic, resulting in polyclonal infected populations. Small interfering RNA (siRNA) transfections (siBCL11B: A673 6.25 nM and TC71 25 nM; siNCOR1: A673 25 nM) were carried out according the manufacturer’s instructions (ThermoFisher). *In vitro* transformation assays were performed by plating 1×10^5^ cells in 2% methylcellulose mixed 1∶1 with cell growth media containing the appropriate antibiotic selection. Endpoint was dictated by the ability to see colonies on control plate (approximately 2–4 weeks; e.g., A673 form colonies faster than TC71), and was internally consistent for all experiments. Plates were sectioned into quadrants, and colonies were counted by eye using an Olympus SZ61 stereomicroscope and manual counter. Growth curves were generated by counting total cells and re-plating 5×10^5^ cells every third day. Student’s T-test was performed on the average three day population difference over fifteen days for each condition. A673 cells were treated with the indicated concentration of the histone deacetylase (HDAC) inhibitor, vorinostat (ChemieTek)or the lysine specific demethylase 1 (LSD1) inhibitor, HCI-2509 [31] for 48 h. A673 cells were treated with the indicated concentration of chaetocin (Sigma-Aldrich), a fungal metabolite that specifically inhibits SUV39H1, or Ex-527, a small molecule that inhibits SIRT1, (Sigma-Aldrich) for 24 h. For ERK detection, A673 cells were infected with empty vector or SPRY1 cDNA and selected with neomycin under normal tissue culture growth conditions. Cells were then trypsinized and plated into tissue culture plates (adherent) or into ultra-low attachment plates (suspension) for 24 h before protein extraction.

### Quantitative Reverse-transcriptase Polymerase Chain Reaction (qRT-PCR)

Total RNA was extracted with an RNAeasy kit (Qiagen). mRNA (15 ng) was quantitated by SYBR green (Bio-Rad) using one-step qRT-PCR with gene specific primers (Table S1). Messenger RNA was reverse-transcribed at 50°C for 10 minutes followed by a 5 minute denaturation at 95°C and then 45 cycles of PCR (95°C for 30 seconds, 57°C for 30 seconds, 72°C for 30 seconds). Fold change was determined using the ΔC_t_ method comparing all samples to the control after normalizing to GAPDH. Student’s T-test was performed using threshold cycle values.

### Immunodetection

Western blots were performed with the following antibodies: FLI1 (Abcam 15289), BCL11B (Abcam 28448), tubulin (Calbiochem CP06), FLAG M2 (Sigma-Aldrich A8592), P-ERK1/2 (Cell Signaling 9106), total ERK1/2 (Cell Signaling 9102).

### RNA Sequencing

RNA from A673 cells transfected with siBCL11B-3, siBCL11B-10 or siControl was extracted with the RNAeasy kit (Qiagen) and treated with DNAse 48 h post-transfection. Libraries for high-throughput sequencing were prepared according to the manufacturer’s instructions (Illumina) and sequenced on the Illumina Hi-Seq with 50 cycles of single end reads. Sequences were aligned to the human genome build hg19. Raw sequence reads can be found in the NCBI SRA #058854. Differential gene expression was determined using the publically available USeq package (useq.sourceforge.net). Significance parameters were set at an FDR of 10% and two-fold change.

Venn diagrams were created using the VennMaster program and comparing BCL11B regulated genes to EWS/FLI repressed genes [9]. GSEA was performed by creating a rank ordered list (most up-regulated genes to most repressed genes) using EWS/FLI knock-down microarray data from A673 cells and TC71 cells [6] and comparing to BCL11B RNAseq data using the GseaPreranked program from the Broad Institute [36].

## Results

### BCL11B Expression is Induced by EWS/FLI

**Figure 1:**
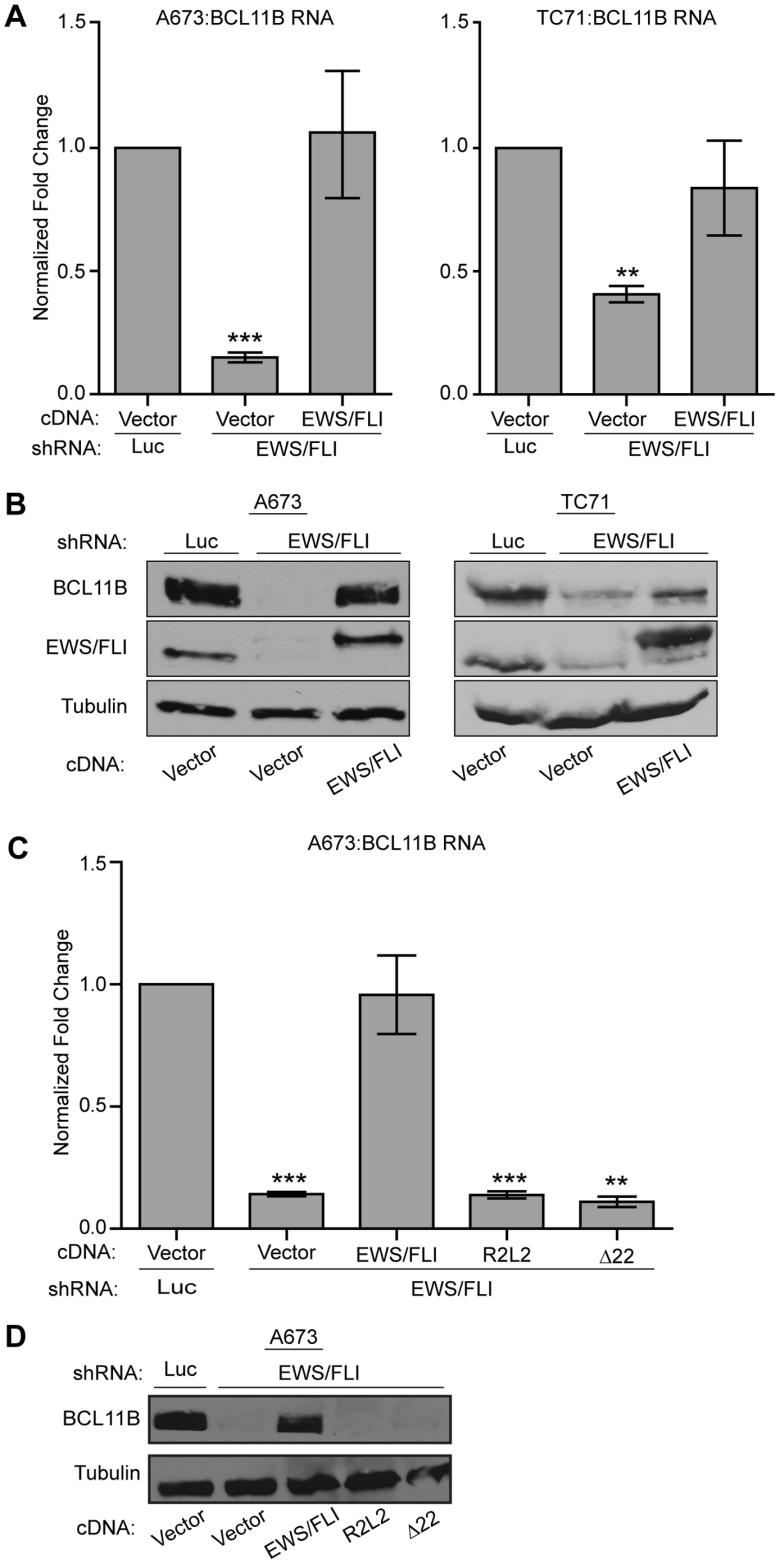
BCL11B is up-regulated by EWS/FLI in Ewing sarcoma cells. A. A673 and TC71 Ewing sarcoma cells were infected with a control shRNA (Luc) or an shRNA targeting EWS/FLI followed by rescue with an empty vector or EWS/FLI cDNA. BCL11B RNA levels were determined by qRT-PCR. Error bars represent standard deviation (SD) of three technical replicates. P-values were determined using a Student’s T-test comparing all conditions to control (Vector+Luc shRNA) (** for p≤0.01, *** for p≤0.001). B. A673 and TC71 cells were infected as in panel A, and protein extracts were Western blotted for BCL11B and EWS/FLI. Tubulin was used as a loading control. C. A673 cells were infected with a control shRNA or shRNA targeting EWS/FLI followed by rescue with EWS/FLI cDNA, or mutants of EWS/FLI containing a DNA biding mutation (R2L2) or an EWS deletion (Δ22). Error bars represent SD of three technical replicates. P-values were determined using a Student’s T-test comparing all conditions to control (Vector+Luc shRNA) (** for p≤0.01, *** for p≤0.001). D. Western blot analysis of BCL11B levels in the same samples shown in panel C. Tubulin was used as a loading control.

BCL11B is highly expressed in Ewing sarcoma cell lines and tumor samples and has been identified as an EWS/FLI up-regulated target in numerous microarray studies [13], [14], [37]. However, the microarray data have never been validated nor has the functional significance of BCL11B in Ewing sarcoma been investigated. Using a knock-down/rescue approach in two different Ewing sarcoma cell lines (A673 and TC71), we found that reduction of EWS/FLI levels via a retroviral shRNA causes a significant reduction in BCL11B RNA and protein expression levels (Figure 1A, B). Furthermore, BCL11B RNA and protein expression are restored when an EWS/FLI cDNA (that is resistant to the RNAi effect) is re-expressed (Figure 1A, B).

EWS/FLI is a modular protein, with transcriptional regulatory functions contributed by the amino-terminus of EWS, and DNA binding contributed by the ETS domain of the FLI portion. We investigated the necessity of these regions in BCL11B regulation by using an EWS deletion construct which retains only the first six amino acids of EWS (Δ22), or a DNA binding double point mutant (R2L2) of EWS/FLI as the rescue construct, respectively (Figure 1C, D; [31]). Neither mutant construct rescued the expression of BCL11B after EWS/FLI knock-down indicating that both the EWS portion (which harbors a strong transcriptional activation domain [38]) and the ETS DNA binding domain are required for the activation of BCL11B.

### BCL11B Expression is Necessary for Maintenance of Transformation

**Figure 2:**
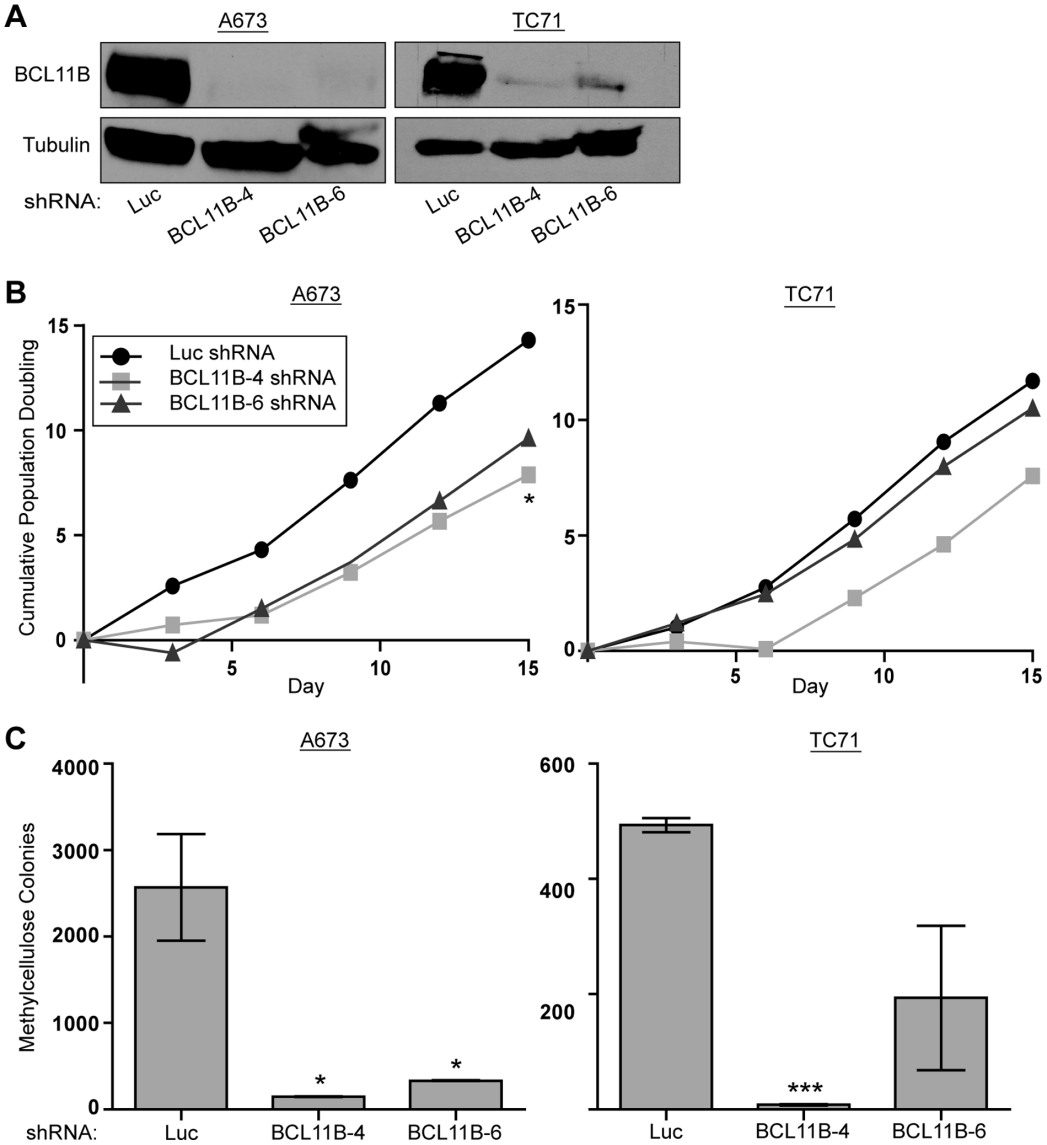
BCL11B is necessary for the maintenance of transformation in Ewing sarcoma cells. A. A673 and TC71 Ewing sarcoma cells were infected with retroviral shRNA constructs targeting BCL11B (BCL11B-4 and BCL11B-6 shRNA), or luciferase (Luc) as a control. BCL11B levels were determined by western blot. Tubulin was used as a loading control. B. Growth rates of A673 and TC71 cells harboring the indicated shRNA retroviral constructs were determined using a 3T5 assay [34]. P-values were determined using a Student’s T-test comparing all conditions to control (Luc shRNA) (* for p≤0.05). C. Anchorage independent growth of control (Luc shRNA) and BCL11B (BCL11B-4 and BCL11B-6 shRNA) knock-down A673 and TC71 cells was assessed by the ability to form colonies in methylcellulose. Error bars represent SD of two technical replicates. P-values were determined using a Student’s T-test comparing all conditions to control (Luc shRNA) (* for p≤0.05, *** for p≤0.001).

We next took a loss of function approach to determine the involvement of BCL11B in maintenance of transformation in Ewing sarcoma. We used anchorage independent growth in methylcellulose as an *in vitro* measure of transformation. A673 and TC71 cells were infected with two different shRNA constructs targeting the 3′UTR of BCL11B (BCL11B-4 and BCL11B-6 shRNA). These shRNAs significantly decreased BCL11B protein levels (Figure 2A). Ewing sarcoma cells with reduced BCL11B expression grew in tissue culture with a slightly reduced growth rate (Figure 2B). However, when these cells were grown in methylcellulose under anchorage-independent conditions, significantly fewer colonies formed as compared to a control knock-down targeting luciferase (Luc shRNA), and correlated with the level of knockdown achieved by each construct (Figure 2C). These results demonstrate that BCL11B is necessary for maintaining the transformed phenotype of Ewing sarcoma cell lines.

### BCL11B Contributes to the EWS/FLI Repressed Gene Signature

**Figure 3:**
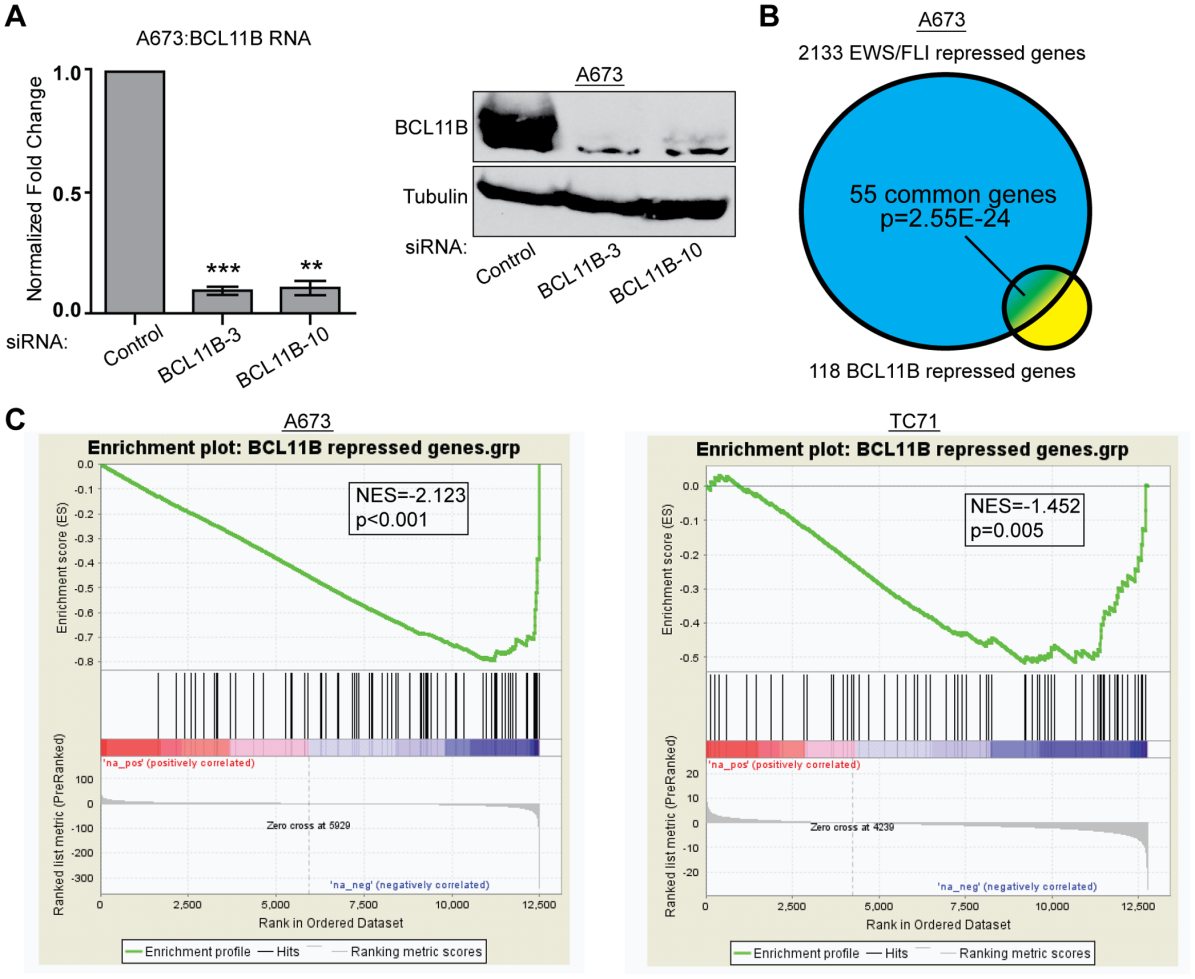
BCL11B represses genes that are part of the EWS/FLI repressed signature. A. qRT-PCR (left) and western blot analysis (right) of A673 cells 48 hours post-transfection with a control siRNA (siControl) or siRNAs targeting BCL11B (siBCL11B-3 and siBCL11B-10). Error bars represent SD of three technical replicates. P-values were determined using a Student’s T-test comparing all conditions to control (** for p≤0.01, *** for p≤0.001). B. Venn diagram analysis shows significant overlap of the 118 gene BCL11B repressed gene set and the 2133 gene EWS/FLI repressed gene set. P-value determined by Chi square analysis. C. GSEA analysis comparing a rank-ordered EWS/FLI gene set (up-regulated genes to the left, down-regulated genes to the right) in A673 and TC71 cells to the BCL11B repressed gene set. NES indicates normalized enrichment scores. P-value determined by permutation testing.

As BCL11B is a transcription factor, we sought to identify the genes it regulates in Ewing sarcoma to gain insight into its function in sustaining tumorigenicity. We performed an RNA-seq experiment comparing transcripts from A673 cells transfected with a control siRNA (siControl) or two different siRNAs targeting BCL11B (siBCL11B-3 and siBCL11B-10) (Figure 3A). We used the Useq package to identify differentially expressed genes with significance cutoffs set to a two-fold change and a false discovery rate (FDR) of 10%. This analysis identified 118 genes down-regulated by BCL11B and 26 genes up-regulated by BCL11B (Table S2). While this analysis does not distinguish direct from indirect targets, this is consistent with BCL11B’s previous characterization as a transcriptional repressor [39].

Venn overlap analysis using genes we found to be repressed by BCL11B and those repressed by EWS/FLI revealed that a significant number of genes were present on both lists. Of the 118 genes repressed by BCL11B, 55 genes were also repressed by EWS/FLI (p = 2.55×10^−24^; Figure 3B). This indicates that the increase in expression of BCL11B mediated by EWS/FLI contributes to the 2133 gene EWS/FLI down-regulated signature. To further establish the relationship between EWS/FLI and BCL11B repressed genes, we used a different unbiased statistical approach by performing Gene Set Enrichment Analysis (GSEA). For this analysis we rank-ordered genes regulated by EWS/FLI from most up-regulated to the most down-regulated, and then identified where on this list the BCL11B repressed genes fell. GSEA produced a significant negative enrichment score (NES) in both A673 and TC71 cells indicating that genes repressed by BCL11B are well-correlated with those most repressed by EWS/FLI (A673 NES = −2.123, p<0.001; TC71 NES = −1.452, p = 0.005; Fig. 3C). In contrast to the down-regulated genes, only two genes from the BCL11B up-regulated gene set overlapped with EWS/FLI up-regulated genes, and were not statistically significant. We therefore focused on the BCL11B repressed genes for the subsequent analyses.

**Figure 4:**
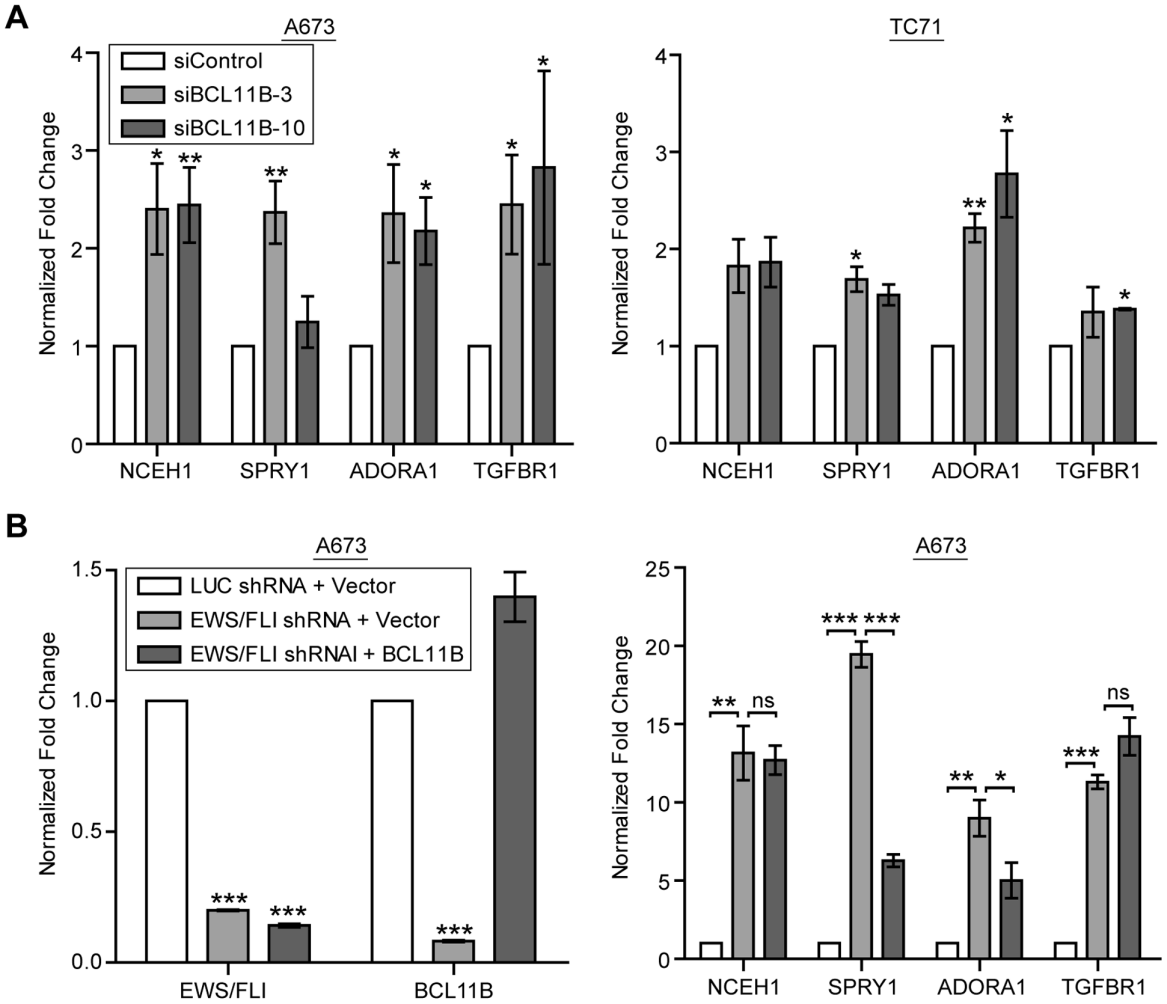
BCL11B mediates repression in Ewing sarcoma cells. A. BCL11B RNA-seq results were confirmed by performing qRT-PCR for the indicated mRNAs on control or BCL11B siRNA transfected A673 cells 48 hours post-transfection. P-values were determined using a Student’s T-test comparing all conditions to control (* for p≤0.05, ** for p≤0.01). B. EWS/FLI was knocked-down using shRNA and rescued with an empty vector or BCL11B cDNA. mRNA levels of EWS/FLI and BCL11B were confirmed by qRT-PCR, and transcript levels of the indicated genes were assessed by qRT-PCR. Error bars represent SD of three technical replicates. P-values were determined using a Student’s T-test comparing all conditions to control (Luc shRNA+Vector) (left panel) or comparing conditions as indicated (right panel) (ns for not significant, * for p≤0.05, ** for p≤0.01, *** for p≤0.001).

Neutral cholesterol ester hydrolase 1 (*NCEH1*), Sprouty1 (*SPRY1*), adenosine A1 receptor (*ADORA1*), and transforming growth factor beta receptor 1 (*TGFBR1*) were found to be repressed by both EWS/FLI and BCL11B in our genome-wide studies. We chose these four genes from the list of fifty-five overlapping genes to validate our findings. We first confirmed our RNA-seq analysis in biologic replicates of A673 cells, as well as TC71 cells, by evaluating these genes with qRT-PCR. We found that when BCL11B levels are reduced by siRNAs, the transcript level of each of the genes increases in both A673 and in TC71 Ewing sarcoma cells (Figure 4A). We then validated the results from the EWS/FLI microarray by knocking down EWS/FLI in A673 cells and again observing an increased expression of these four genes (Figure 4B).

We next wanted to determine if the up-regulation of BCL11B by EWS/FLI was solely responsible for this repression. To test this, we used shRNA targeting EWS/FLI in A673 cells (which decreases BCL11B expression) and re-expressed the BCL11B cDNA. We found that BCL11B expression was able to partially rescue the increase in expression of SPRY1 and ADORA1, but not NCEH1 and TGFBR1 (Figure 4B). This suggests that BCL11B has some repressive activity against SPRY1 and ADORA1 independent of EWS/FLI; however, full repression of these genes, as well as repression of NCEH1 and TGFBR1, requires more complex regulation, likely including other genes regulated by EWS/FLI.

### BCL11B Repression is Mediated by the NuRD Complex

BCL11B has been characterized as a transcriptional repressor in many cellular contexts. This repression is mediated by interactions with various co-repressors including the nucleosome remodeling and histone deacetylase complex (NuRD) [40], [41], suppressor of variegation 3–9 homolog 1 (SUV39H1) [42], [43], and sirtuin1 (SIRT1) [44]. We tested the necessity of these three co-repressors in BCL11B-mediated repression in Ewing sarcoma cells by shRNA knock-down for chomodomain helicase DNA binding protein 4 (CHD4), the core component of the NuRD complex, or chemical inhibitors targeting SUV39H1 or SIRT1. We found that the small molecule inhibitor of SIRT1, Ex-527, had no effect on the expression of NCEH1, SPRY1, ADORA1, or TGFBR1 (Figure S1A). Chaetocin, a fungal metabolite that specifically inhibits SUV39H1, was cytotoxic to A673 cells at concentrations lower than the IC50 (800 nM) for inhibition of SUV39H1 (data not shown). Even at these lower concentrations, it significantly reduced the expression of BCL11B (Figure S1B), and so prevented us from drawing any conclusions about the involvement of SUV39H1 in BCL11B mediated repression in Ewing sarcoma.

**Figure 5:**
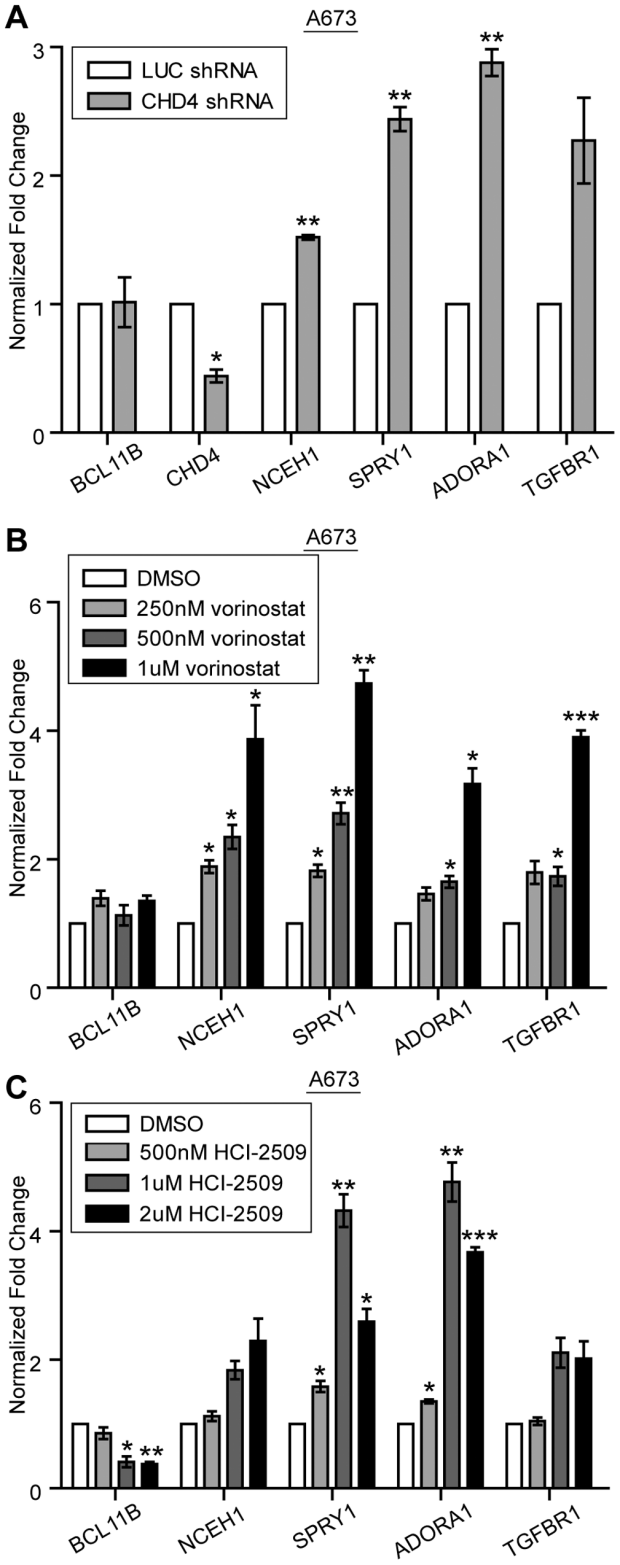
BCL11B mediates transcriptional repression via the NuRD complex. A. shRNA knock-down of CHD4 in A673 cells results in the up-regulation of BCL11B repressed genes as measured by qRT-PCR. B. A673 cells treated with the indicated dose of vorinostat for 48 hours results in the dose-dependent increase of the indicated BCL11B repressed genes as measured by qRT-PCR. C. qRT-PCR of A673 cells treated with the indicated dose of the LSD1 inhibitor, HCI-2509, for 48 hours. Error bars represent SD of three technical replicates. P-values were determined using a Student’s T-test comparing all conditions to control (Luc shRNA (A) or DMSO (B,C)) (* for p≤0.05, ** for p≤0.01, *** for p≤0.001).

In contrast to SIRT1 and SUV39H1, we found that targeting of the core NuRD component CHD4, using a retroviral shRNA in A673 cells, increased expression of NCEH1, SPRY1, ADORA1, and TGFBR1 (Figure 5A), while levels of BCL11B were unchanged. In addition to its nucleosome remodeling activity, the NuRD complex also contains class I histone deacetylases (HDACs). To test the necessity of HDACs in this repression, A673 cells were treated with the HDAC inhibitor vorinostat. After 48 hours of treatment, all four of the BCL11B target genes we identified were derepressed in a dose-dependent manner (Figure 5B). In addition, a more recently identified member of the NuRD complex, lysine specific demethylase 1 (LSD1) [45], was tested for its involvement in this repression. When A673 cells were treated with a recently-described small molecule inhibitor of LSD1, HCI-2509, the expression of these four genes was increased with a dose of 1 uM, the IC50 for this compound. It must be noted that HCI-2509 also decreased the expression of BCL11B at this dose (Figure 5C), rendering interpretation of this result difficult. As a control, we used siRNA targeting NCOR1, a co-repressor that also interacts with HDACs and has not been shown to effect BCL11B-mediated repression. Knock-down of NCOR1 had no effect on the BCL11B repressed genes (Figure S1C).

### Re-expression of SPRY1 Limits Transformation in Ewing Sarcoma Cells

**Figure 6:**
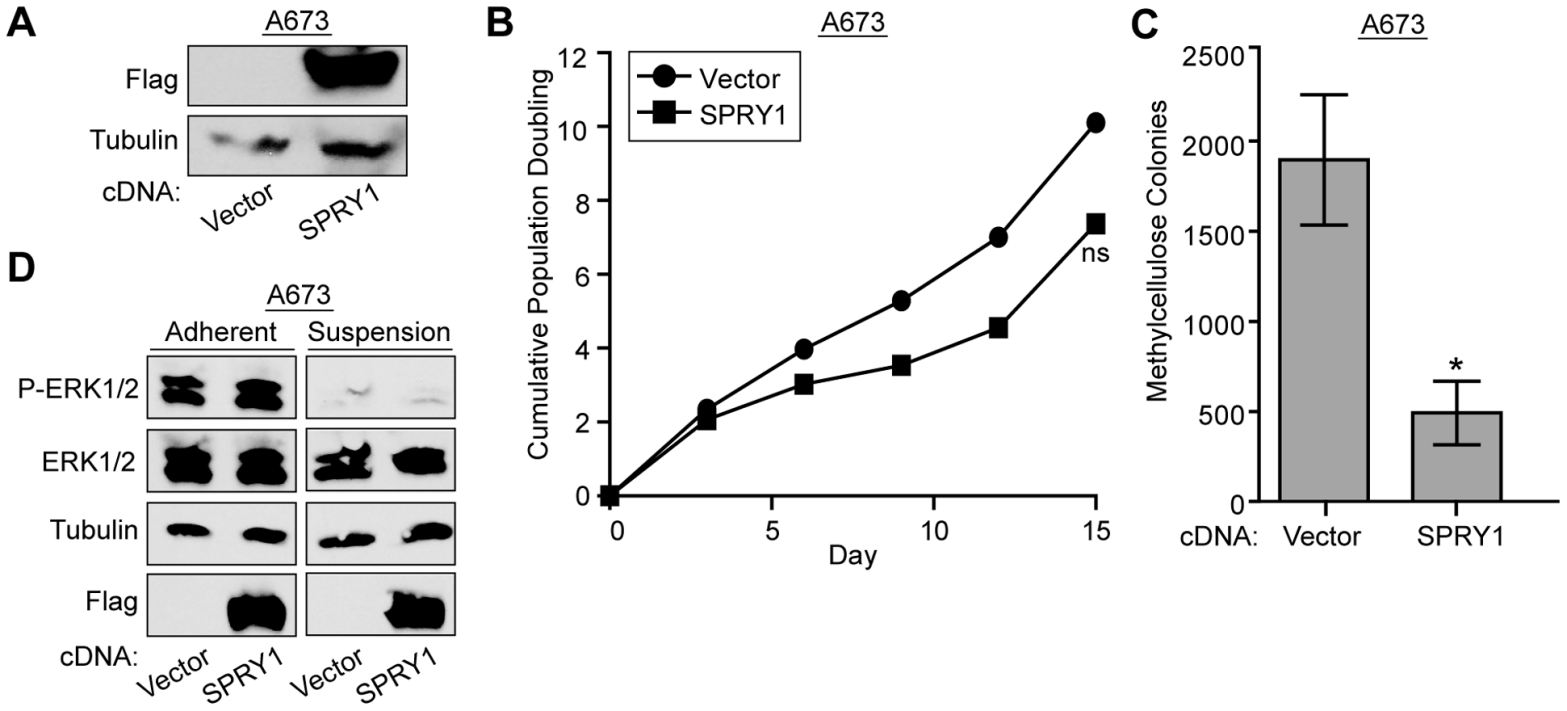
Re-expression of SPRY1 limits the transformation potential of Ewing sarcoma cells. A. Western blot shows expression of 3xFLAG SPRY1 construct in A673 cells. B. Growth rates of A673 cells expressing 3xFLAG SPRY1 were determined using a 3T5 assay. P-values were determined using a Student’s T-test (ns for not significant).C. Anchorage independent growth of A673 cells expressing 3xFLAG SPRY1 was assessed by the ability to grow in methylcellulose. Error bars represent SD of two technical repeats. P-values were determined using a Student’s T-test (* for p≤0.05). D. Western blot shows levels of phosphorylated and total ERK1/2 when A673 cells are grown under adherent or suspension conditions in the presence or absence of 3xFLAG SPRY1 cDNA. Tubulin is used as a loading control and flag shows 3xFLAG SPRY1 expression.

To further investigate the involvement of BCL11B mediated repression in the maintenance of *in vitro* transformation, we asked whether re-expression of one such BCL11B-repressed gene, *SPRY1*, could affect colony growth in methylcellulose. SPRY1 has multiple potential functions, but is best known as a negative regulator of the RAS/MAPK signaling pathway [46]. Because RAS/MAPK signaling contributes to cell growth and proliferation, and is active in Ewing sarcoma cells [47], it seemed plausible that re-expression of an inhibitor of this pathway, SPRY1, could reduce cell proliferation or transformation. We tested this by expressing a 3xFLAG-tagged SPRY1 cDNA in the A673 Ewing sarcoma cell line (Figure 6A) and performing growth curves and *in vitro* transformation assays. We found that SPRY1 expression had a minor effect on cell proliferation in tissue culture (Figure 6B). However, it significantly limited the ability of these cells to form colonies under anchorage independent growth conditions (Figure 6C). Surprisingly, this effect did not seem to be mediated via inhibition of RAS/MAPK signaling, as phosphorylation of the downstream effectors, ERK1/2, remained unchanged in cells re-expressing SPRY1 (Figure 6D). When cells were grown in ultra-low attachment plates for 24 h, P-ERK1/2 levels drastically decreased irrespective of SPRY1 expression (Figure 6D). This suggests that SPRY1 mediates inhibition of anchorage-independent growth via an alternate, RAS/MAPK-independent, pathway.

## Discussion

We have shown that EWS/FLI is responsible for the expression of BCL11B in Ewing sarcoma cells. The dramatic decrease in BCL11B expression levels with EWS/FLI knock-down suggests that BCL11B is not normally expressed in the Ewing sarcoma cell of origin. BCL11B is, however, necessary for the proper development of many organs including the brain [21], [22], [24], skin [19], and teeth [20], as well as for terminal differentiation of T-cells [18], [23]. This allows for the possibility that in the likely primitive Ewing sarcoma cell of origin, the BCL11B promoter has an open chromatin structure that permits the strong activation of BCL11B following the formation of the translocation. At this time we consider BCL11B an indirect target of EWS/FLI as EWS/FLI did not show binding to the BCL11B promoter in our ChIP-chip data set [48]. Furthermore previously defined EWS/FLI binding sites – GGAA repeats and ETS consensus sites – are absent from the BCL11B promoter/enhancer region. There are however variant ETS sites in BCL11B’s promoter/enhancer region that may allow for EWS/FLI binding and warrant further investigation.

This EWS/FLI-dependent up-regulation of BCL11B is necessary for the maintenance of the transformed phenotype in Ewing sarcoma cell lines *in vitro*. This oncogenic capacity is in stark contrast to its tumor inhibitory function in leukemia and lymphoma – the cancers in which BCL11B have mainly been studied [49]. Mutations or deletions of the *BCL11B* gene are found in 9–16% of human T-ALL [25], [26] where it is thought to be a haploinsufficient tumor suppressor. Loss of one allele via the involvement of BCL11B in translocations in this malignancy as well as distinct mutations often found in the zinc finger region [25] may contribute to the oncogenic process in part by preventing differentiation. Moreover in a mouse model of thymic lymphoma, spontaneous homozygous deletions and point mutations occurred in Bcl11b. This group also found that ectopic expression of Bcl11b in HeLa cells suppressed cell growth [27]. BCL11B has not been widely studied in the context of solid malignancies and is not expressed in many [50].

The differing function of BCL11B in Ewing sarcoma does not appear to be due to a unique transcriptional activity in this tumor: BCL11B acts mainly as a transcriptional repressor in Ewing sarcoma, and thus acts similarly to what has been demonstrated in other cellular contexts. However, BCL11B regulates a unique set of genes in the A673 cellular background in comparison to other cell types in which it has been studied. For example, BCL11B has been implicated in cell cycle progression by directly repressing the cell cycle inhibitors p21WAF1 [43] and p57KIP2 [41] in microglial cells and SK-N-MC (which were originally characterized as a neuroblastoma cell line, but are in fact a Ewing cell line) cells, respectively. We were able to confirm downregulation of p57KIP2 transcript levels by BCL11B in SK-N-MC cells by qRT-PCR; however this was not observed in A673 or TC71 cells (ETW unpublished observation). At this time, we do not understand the differences among these cellular contexts that account for this discrepancy. Nonetheless, this discrepancy across Ewing sarcoma cell lines suggests that inhibition of p57KIP2 is not a central feature of BCL11B function in this tumor type. In developing T-cells BCL11B represses genes that allow for a more primitive state thus contributing to the differentiation process [23]. These classes of genes were not observed in our genome wide analysis of BCL11B regulated genes in A673 cells. Our data suggests that BCL11B contributes to the overall EWS/FLI repressed gene signature in Ewing sarcoma, and that the repression of a subset of these genes may be necessary for the transformed phenotype. EWS/FLI directly represses approximately 5% of the total EWS/FLI repressed genes [31]. This allows for a model where EWS/FLI up-regulates the expression of transcriptional repressors, such as BCL11B, which then indirectly account for the repression of the remaining 95% of the EWS/FLI down-regulated genes.

BCL11B facilitates transcriptional repression by recruiting a variety of chromatin modifying enzymes to the promoters of genes. BCL11B physically interacts with the histone methyltransferase SUV39H1 [42], [43], the histone demethylase LSD1 [51], histone deacetylases HDAC1 and HDAC2 [42], the class III histone deacetylase, SIRT1 [44], as well as the NuRD co-repressor complex [40], [41]. BCL11B likely utilizes these interactions to mediate repression in both a cell-type specific and a promoter-specific fashion. We have shown that the chromatin remodeling activity of the NuRD co-repressor complex participates in the repression of the four BCL11B repressed genes investigated in this study. Our data further suggest that vorinostat-repressible HDAC activity is involved in this repression. These data are consistent with vorinostat inhibition of NuRD-associated HDACs, but inhibition of non-NuRD associated HDACs may also play a role. We have also demonstrated that the LSD1 inhibitor, HCI-2509, increases the expression of these genes. The mechanism for HCI-2509 is unclear due to the fact that BCL11B levels are also somewhat reduced. This small molecule has recently been shown to de-repress some EWS/FLI directly repressed target genes and show specific toxicity for Ewing sarcoma cells [31]. The data presented here further demonstrate that HCI-2509 is able to reverse at least a portion of the EWS/FLI regulated gene signature which provides a possible mechanism for the toxicity to Ewing sarcoma cells.

We have not investigated the direct or indirect nature of this BCL11B mediated repression. *In vitro* studies have identified a GC-rich BCL11B binding site [39]; however, a genome-wide analysis of BCL11B binding *in vivo* [52] failed to confirm the *in vitro* findings. Thus, a *bona fide* BCL11B consensus site remains elusive. Without this information we were unable to inspect the promoters of our BCL11B regulated genes to identify potential direct targets. We did however perform motif enrichment analysis using the MEME suite [53] on the BCL11B repressed gene list to identify any enriched sequence motifs. This analysis failed to show any significant sequence enrichment (ETW, unpublished observations). This may be expected due to the mixed set of directly and indirectly regulated genes.

We have shown that the re-expression of the BCL11B repressed gene, *SPRY1*, reduces the transformation potential of Ewing sarcoma cells. This further implicates BCL11B mediated repression as an important contributor to the repressed gene signature in Ewing sarcoma cells. At this time the mechanism involved in SPRY1’s ability to reduce transformation remains unclear, although it does not appear to be acting in its classic role by inhibiting RAS/MAPK signaling. Spry1 is also known to inhibit phospholipase C (PLC) activation, and during *Xenopus* mesoderm development, *Xtsprouty* inhibits the PLC pathway while still allowing for RAS/ERK signaling. SPRY1 may be impinging on an alternate growth factor signaling pathway or perform a novel function in the context of a Ewing sarcoma cell.

A recurring theme in Ewing sarcoma is alteration in the expression levels of important developmental genes. Various pathways involved in proper development and differentiation have been disrupted by EWS/FLI in Ewing sarcoma – sonic hedgehog [10], [54], transforming growth factor beta (TGFB) [11], [31], and WNT [55], among others. The timely expression of BCL11B during the development of many cell types is crucial for proper differentiation. Here we have shown the aberrant expression of BCL11B in Ewing sarcoma cell lines represses a subset of the EWS/FLI repressed gene signature and contributes to the transformed phenotype.

## Supporting Information

Figure S1 **Investigating mechanisms of BCL11B mediated repression.** A. qRT-PCR from A673 cells treated with the indicated dose of the SIRT1 inhibitor, Ex-527, for 24 hours. B. qRT-PCR data from A673 cells treated with the indicated dose of the SUV39H1 inhibitor, Chaetocin, for 24 hours. C. qRT-PCR data from A673 cells transfected with siRNA targeting NCOR1 (siNCOR1) or control (siControl) for 48 hours. Error bars represent SD of three technical replicates. P-values were determined using a Student’s T-test comparing all conditions to control (DMSO (A,B) or siControl (C) (* for p≤0.05, ** for p≤0.01, *** for p≤0.001).
(TIF)

Table S1 **Primer Sequences.**
(DOCX)

Table S2 **BCL11B RNAseq Data with FDR 10% and 2 fold change.**
(XLSX)
