# Supplementary figures and images for: BCL11B Is Up-Regulated by EWS/FLI and Contributes to the Transformed Phenotype in Ewing Sarcoma
Source: PLoS One. 2013 Mar 19;8(3):e59369. doi: 10.1371/journal.pone.0059369 (PMC3601955; doi:10.1371/journal.pone.0059369)

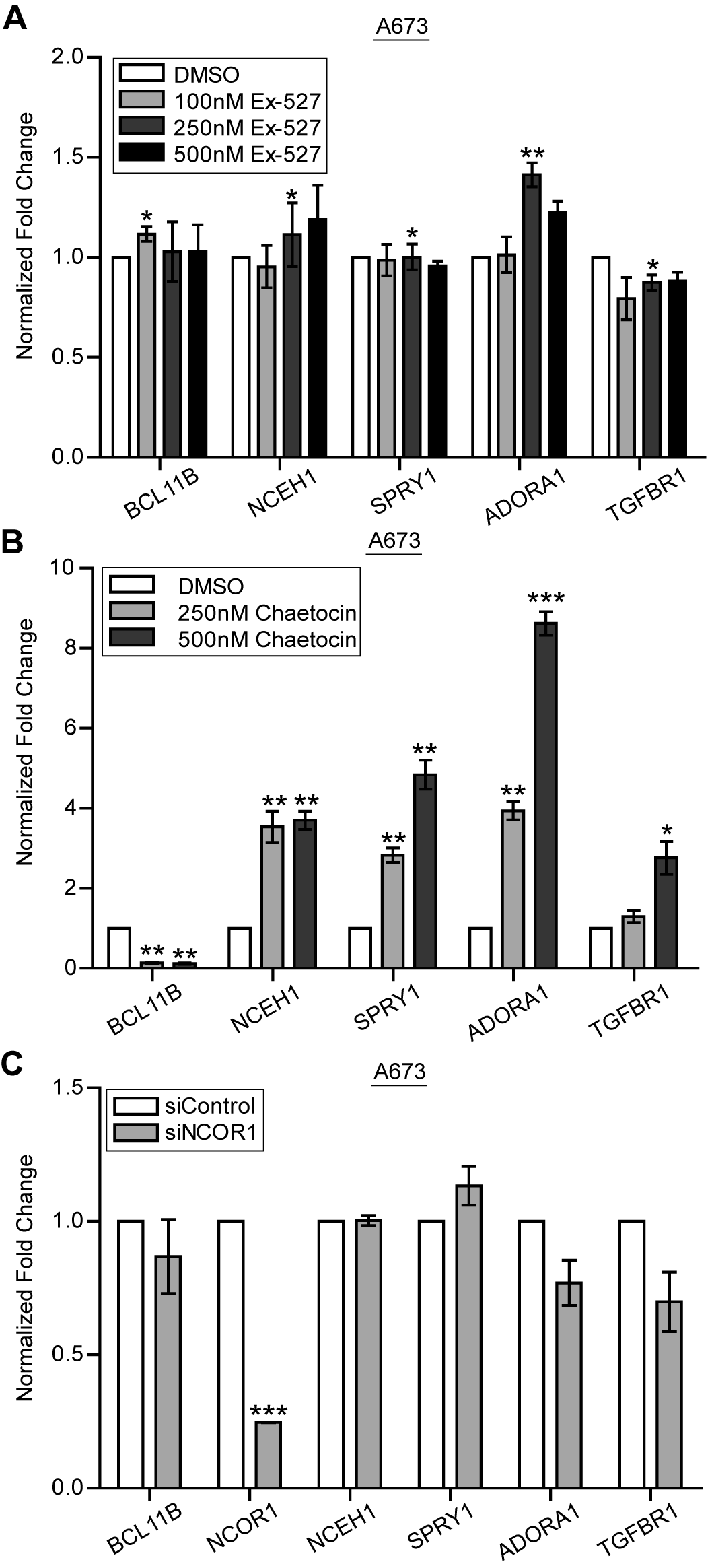

Supplement: Figure S1 — Investigating mechanisms of BCL11B mediated repression. A. qRT-PCR from A673 cells treated with the indicated dose of the SIRT1 inhibitor, Ex-527, for 24 hours. B. qRT-PCR data from A673 cells treated with the indicated dose of the SUV39H1 inhibitor, Chaetocin, for 24 hours. C. qRT-PCR data from A673 cells transfected with siRNA targeting NCOR1 (siNCOR1) or control (siControl) for 48 hours. Error bars represent SD of three technical replicates. P-values were determined using a Student’s T-test comparing all conditions to control (DMSO (A,B) or siControl (C) (* for p≤0.05, ** for p≤0.01, *** for p≤0.001). (TIF) [file pone.0059369.s001.tif]
