# Supplementary material for: BCL11B Is Up-Regulated by EWS/FLI and Contributes to the Transformed Phenotype in Ewing Sarcoma
Source: PLoS One. 2013 Mar 19;8(3):e59369. doi: 10.1371/journal.pone.0059369 (PMC3601955; doi:10.1371/journal.pone.0059369)
Supplement: Table S1 — Primer Sequences. (DOCX) [file pone.0059369.s002.docx]

Table S1 Primer Sequences:

shRNA sequences:

| iBCL11B-4 | 5’CTGAATTACTCTAAGCAAATTCAAGAGATTTGCTTAGAGTAATTCAG3’ |
| --- | --- |
| iBCL11B-6 | 5’GGAAATGGCCCGACAGCATTTCAAGAGAATGCTGTCGGGCCATTTCC3’ |
| iCHD4 | 5’CTGCCAGGCTTGAAGAAAATTCAAGAGATTTTCTTCAAGCCTGGCAG3’ |

qRT-PCR primers:

| Gene | Forward | Reverse |
| --- | --- | --- |
| BCL11B | 5’GCTCATCACCCCAGAGGCTGAC3’ | 5’GTGCAAATGTAGCTGGAAGGCTCATC3’ |
| NCEH1 | 5’GCACAGCAAGTGAGTAACCTGATCC3’ | 5’GCACTTGCCAAGGCCCAGC3’ |
| SPRY1 | 5’C CGAAAAGGATTTCAGATGCATGCC3’ | 5’GGTCTTTTCACCACCGAAGGCC3’ |
| ADORA1 | 5’CCTCTCCGGTACAAGATGGTGG3’ | 5’CCAGGTAGATGAGGACCATGAGG3’ |
| TGFBR1 | 5’GGTCCTGTGGAGCTGGCAGC3’ | 5’GCAGTGGTAAACCTGATCCAGACC3’ |
| CHD4 | 5’CACCCCACCCAGAAAATGAAGAGG3’ | 5’CCGGCATAAGAGCATACGCTCC3’ |
